# Breast cancer in Africa: an extensive surgical burden of paramount importance – letter to the editor

**DOI:** 10.1097/JS9.0000000000000253

**Published:** 2023-02-16

**Authors:** Andrew A. Wireko, Jack Wellington, Favour T. Adebusoye, Pearl O. Tenkorang, Amal O. Ahmad, Toufik Abdul-Rahman

**Affiliations:** a Sumy State University, Sumy, Ukraine; b Cardiff University School of Medicine, Cardiff University, Wales, UK; c University of Ghana Medical School, Accra, Ghana

*Dear Editor*,

One form of cancer that begins in the breast tissue is breast cancer (BC). Due to its greater mortality rate than most other malignancies and its annual prevalence of 2.1 million women, BC is a serious health problem for women^1^. In 2018, there were ∼627 000 BC-related deaths, with sub-Saharan Africa accounting for 15% of all cancer-related deaths^1^. Furthermore, according to the 2020 Global Cancer Observatory (GLOBOCAN), 186 598 BC cases and 85 787 related deaths were recorded in Africa^1^. BC is expected to more than double in many African countries by 2050, indicating that its incidence and mortality are rising disproportionately in Africa^1^.

Africa continues to lag far behind, despite major improvements in BC management in the west. In contrast to other high-income countries, BCs in Africa are typically discovered and diagnosed in advanced stages. For effective treatment of BC, a multidisciplinary strategy involving surgery, chemotherapy and other methods is warranted. However, surgery is the sole choice in the majority of African countries because all other supplementary therapy options are either unavailable or prohibitively expensive.

The two most popular surgical treatments for BCs are mastectomy and breast conservative surgery. Due to the high prevalence of advanced-stage BC presentations, primarily as a result of insufficient diagnostic and screening systems, mastectomy is frequently utilised in most African countries. A study conducted in sub-Saharan Africa discovered that breast conservative surgeries were performed on 16–26% of patients, and mastectomy was performed on 64–67% of BC patients^2^.

In the context of perioperative outcomes, an examination of postoperative complications in 113 BC resection surgery patients in Rwanda between 2007 and 2016 revealed a higher proportion of postoperative complications^3^. Early postoperative complications affected 56.6% of BC patients, while late complications affected 60.2%^3^. Pain, seroma, haematoma, operative wound infection, embolism and delayed healing are all common postoperative complications of African BC surgeries^3^. A study in Burkina Faso discovered 28.95% of BC surgery postoperative complications, while a similar study in Nigeria discovered 14.5%^4^,^5^. The increased frequency of early postsurgical pain can be attributed to insufficient or nonavailability of opioids to reduce pain^3^.

Another study of mastectomies performed at a Nigerian teaching hospital between 2012 and 2019 found that 36% of patients required perioperative blood transfusions due to excessive bleeding during surgery^6^. The postoperative problems of 19 of the 28 patients who received blood transfusions deteriorated, confirming the notion that blood transfusion is substantially linked to postoperative morbidity^6^. Furthermore, Ayoade *et al*.^6^ discovered two complications that contribute significantly to the length of hospital stay of BC patients following up surgery: flap necrosis (9.1%) and wound infection (6.5%). Moreover, 7.8% of the patients experienced a local recurrence, mostly due to a lack of follow-up for radiotherapy and other postoperative care^6^.

There is a scarcity of surgeons trained to perform BC surgeries such as mastectomies, complete axillary node dissection, and breast reconstruction procedures in many parts of Africa. Although the number of BC cases requiring surgery has significantly increased, Africa only has 0.5 surgeons and 0.1 anaesthetists per 100 000 inhabitants^7^. For instance, Rwanda has no surgical oncologists who can treat patients with BC and just 15 general surgeons. Similar to Niger, just two institutions in this nation have surgical oncologists despite the high frequency of the disease there^8^. Despite breast reconstruction surgery becoming an increasingly important aspect of BC management, particularly in high-income countries, breast reconstruction surgery is almost completely unavailable in many African nations due to a variety of factors such as high costs and a lack of reconstructive surgeons, among many others.

Modern hospitals, specialised facilities, and enhanced technology are necessary for the effective surgical management of cancers. These are scarce in the majority of African nations, especially in rural or outlying areas. For instance, Malawi only has two BC surgery centres that serve a sizable population^9^. Only seven hospitals in the Gambia currently provide BC surgery, where 40% of BC patients have access to these clinics, according to a study conducted. Despite this troubling situation, BC has severely unequal access to these few facilities^10^.

Poor BC surgical outcomes are caused in large part by financial hardships and poverty in many African nations. Studies show that a significant portion of patients in Uganda and Nigeria (86.4%) said that the expense of their care was the main barrier preventing them from seeking further treatment. Surgery is extremely expensive for people without insurance or who cannot pay out-of-pocket expenses. They are unable to get the appropriate medical care as a result^8^.

The accessibility of surgical therapy for BC in Africa is significantly influenced by cultural and societal variables. Patients are discouraged from seeking surgical intervention since the condition and its treatment are stigmatised in some countries. According to studies performed in Ghana, Eritrea and Nigeria, a sizable fraction of BC patients who are considering surgery first consult with alternative healthcare providers such as traditional healers, prayer centres and herbalists. These studies demonstrate that 12.2–38.4% of patients engage in such behaviour. Patients’ decisions to undergo BC surgery are greatly influenced by traditional ideas concerning sickness and treatment^8^.

For Africa to catch up to the world’s BC surgical capability, much effort remains. Expanding BC and other oncological hospitals and facilities could achieve this. Promoting the growth of BC clinics could make it easier for BC patients to get specialised treatment, especially in rural or distant places. It is imperative to give top priority to initiatives that assist the opening of new clinics or the growth of existing ones. Among these unique advancements could be investments in telemedicine and remote consultation services, like telemedicine. Patients could communicate in real-time with surgeons and other healthcare professionals across the nation or continent with the aid of telemedicine and other remote consultation services. In rural or remote places where access to care may be constrained, this could be exponentially effective.

Furthermore, improving financial support for surgical training programmes would be a major improvement for African countries. Financially supporting programmes for surgical training may contribute to an expansion in the pool of general, breast and plastic surgeons accessible to treat BC cases. To meet the surgical demands of the continent, educational initiatives should concentrate on training indigenous surgeons, especially in rural areas where needs are unmet. Moreover, education may be used to substantially lower the frequency of BCs in Africa through civilian education initiatives and programmes.

## Ethical approval

Ethical approval is not required.

## Sources of funding

No funding was sourced.

## Author contribution

A.A.W.: conceptualisation, writing and editing; J.W., F.T.A., P.O.T., A.O.A. and T.A.-R.: writing and editing. All authors agreed to the final manuscript submission.

## Conflicts of interest disclosure

There were no conflicts of interest.

## Guarantor

Andrew Awuah Wireko.

## Data availability

The data that support the findings of this study are available from the corresponding author, Andrew Awuah Wireko, upon reasonable request. The data are not p ublicly available since this could compromise the privacy of research participants.
